# Inflammation: a way to understanding the evolution of portal hypertension

**DOI:** 10.1186/1742-4682-4-44

**Published:** 2007-11-13

**Authors:** María-Angeles Aller, Jorge-Luis Arias, Arturo Cruz, Jaime Arias

**Affiliations:** 1 Surgery I Department. Medical School, Complutense University, 28040 Madrid, Spain; 2 Psychobiology Laboratory, School of Psychology, University of Oviedo, Asturias, Spain; 3 General Surgery Department, Virgen de la Luz General Hospital, 16002 Cuenca, Spain

## Abstract

**Background:**

Portal hypertension is a clinical syndrome that manifests as ascites, portosystemic encephalopathy and variceal hemorrhage, and these alterations often lead to death.

**Hypothesis:**

Splanchnic and/or systemic responses to portal hypertension could have pathophysiological mechanisms similar to those involved in the post-traumatic inflammatory response.

The splanchnic and systemic impairments produced throughout the evolution of experimental prehepatic portal hypertension could be considered to have an inflammatory origin. In portal vein ligated rats, portal hypertensive enteropathy, hepatic steatosis and portal hypertensive encephalopathy show phenotypes during their development that can be considered inflammatory, such as: ischemia-reperfusion (vasodilatory response), infiltration by inflammatory cells (mast cells) and bacteria (intestinal translocation of endotoxins and bacteria) and lastly, angiogenesis. Similar inflammatory phenotypes, worsened by chronic liver disease (with anti-oxidant and anti-enzymatic ability reduction) characterize the evolution of portal hypertension and its complications (hepatorenal syndrome, ascites and esophageal variceal hemorrhage) in humans.

**Conclusion:**

Low-grade inflammation, related to prehepatic portal hypertension, switches to high-grade inflammation with the development of severe and life-threatening complications when associated with chronic liver disease.

## Introduction

Portal hypertension is a clinical syndrome defined by a pathological elevation of blood pressure in the portal system [1-3]. It manifests clinically as ascites, portosystemic encephalopathy and variceal hemorrhage, and often leads to death [4].

Nowadays, a fundamental objective of both experimental and clinical research is the knowledge of the molecular mechanisms underlying this complex syndrome. However, the integration of these pathophysiological mechanisms in trying to understand their possible meaning is also of great interest.

Knowing the final meaning of the alterations associated with portal hypertension could help to understand the meaning of the mechanisms involved in its production and maintenance. Therefore, it would be justified to speculate about the hypothetical purpose of the splanchnic and systemic responses to portal hypertension [5] since the keys for understanding the true meaning of the diverse etiopathogenic factors involved in its production could be obtained.

We have, therefore, proposed an inflammatory etiopathogenic hypothesis of the complications of portal hypertension [6]. If so, the inflammation of the splanchnic system could be the basic mechanism that drives the essential nature of the different complications of portal hypertension. Likewise, inflammation could facilitate the integration of the pathophysiological mechanisms involved in the different complications of portal hypertension [5,6].

As science grows more complex it is also converging on a set of unifying principles that link apparently disparate diseases through common biological pathways and therapeutic approaches [7]. Thus research tactics and strategies may become very similar across diseases [7,8]. In this way, by integrating the mechanisms that govern the inflammatory response with the complications related to the evolution of portal hypertension could enrich their pathogenic knowledge.

## The inflammatory response to injury by mechanical energy

Mechanical energy represents an old stimulus that causes, by cell mechanotransduction, responses considered both physiological and pathological [9]. Specifically, this type of energy can stimulate the endothelium which, owing to its strategic position, plays an exceedingly important role in regulating the vascular system by integrating diverse mechanical and biochemical signals and by responding to them through the release of vasoactive substances, chemokines, cytokines, growth factors and hormones [9-11].

Mechanical energy is obviously involved in the etiopathology of mechanical traumatisms and can produce either local or generalized acute inflammation [12-15].

**Figure 1:**
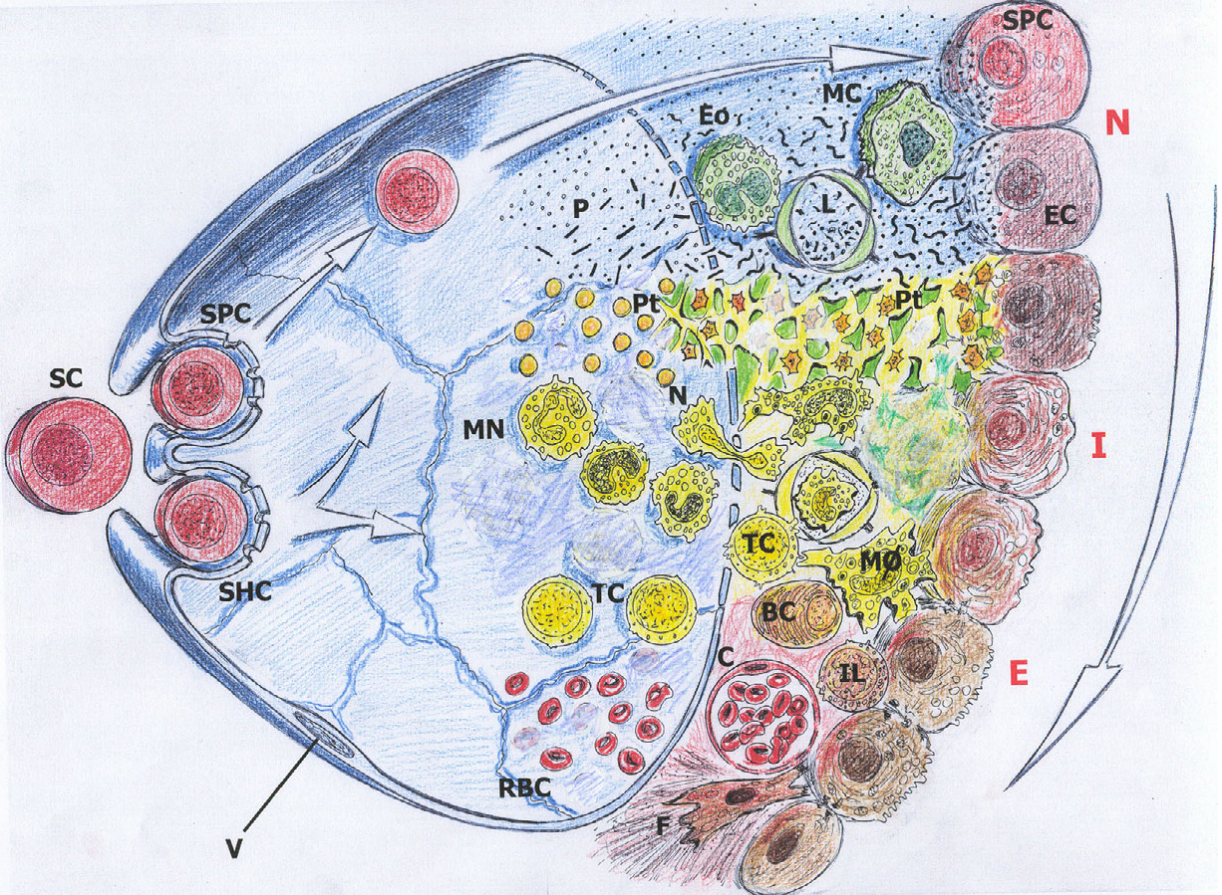
**Post-traumatic acute inflammatory response**. During the first, immediate or *nervous phase *(N) of the acute inflammatory response ischemia-revascularization is produced with edema and oxidative stress. In the second, intermediate or *immune phase *(I) coagulation and infiltration of the interstitium is produced by leukocytes and bacteria. During the nervous and immune phases lymphatic circulation plays a major role. In the third, final or *endocrine phase *(E), nutrition mediated by the blood capillaries is established due to angiogenesis. SC: Stem cell; SPC: Stem pleiotropic cell; SHC: Stem hematopoietic cell; Eo: Eosinophil; MC: Mast cell; EC: Epithelial cell; P: Plasma; Pt: Platelets; L: Lymph; MN: Monocytes; N: Neutrophils; TC: T cells; MØ: Macrophage; BC: B cells; IL: Intraepithelial lymphocyte; RBC: Red blood cells; C: Capillary; F: Fibroblast; V: postcapillar venule

The successive pathophysiological mechanisms that develop in the interstitial space of tissues when they undergo acute post-traumatic inflammation are considered increasingly complex trophic functional systems for using oxygen [12-15]. Although their length would be apparently different, the hypothetical similarity of the local and systemic responses to mechanical injury could be attributed to the existence of a general response mechanism to the injury in the body that is based on the successive and predominant expression of the nervous, immune and endocrine pathological functions [12-14] (Figure 1).

The nervous or immediate functional system presents ischemia-reperfusion and edema, which favor nutrition by diffusion through injured tissue. This trophic mechanism has a low energy requirement that does not require oxygen (ischemia) or in which the oxygen is not correctly used, with the subsequent development of reactive oxygen and nitrogen species (ROS/RNS) (reperfusion). The intense activation of the hypothalamic-pituitary-adrenal axis and the adrenomedullary system with glucocorticoids secretion, the release of epinephrine into the circulation and the activation of the renin-angiotensin-aldosterone system, makes the selective accumulation of these substances in the interstitial space of the tissues and organs that suffer ischemia-reperfusion possible because their endothelial permeability is increased [12,14]. Disturbances in organ blood flow, by vasomotor alterations and systemic redistribution of the blood flow, are suggested to play a pivotal role in the development of progressive organ dysfunction. Furthermore, the splanchnic organs are considered to be one of the key components in the pathogenesis of multiple organ failure [16,17] (Figure 1).

The immune or intermediate functional system activates the coagulation-fibrinolisis system and produces infiltration of the injured tissue by inflammatory cells, especially by leukocytes and bacteria. Also, the immune cell residents in the interstitial space of the affected tissues and organs are activated. Hence, symbiosis of the inflammatory cells and bacteria for extracellular digestion by enzyme release (fermentation) and intracellular digestion by phagocytosis, could be associated with a hypothetical trophic capacity [12-14]. Improper use of oxygen persists in this immune phase [14]. Also during this phase the lymphatic circulation continues to play an important role [14,15]. Macrophages and dendritic cells migrate to lymph nodes where they activate T lymphocytes, which could be another link in the leukocytic trophic chain [18]. Furthermore, in this phase an Acute Phase Response (APR), that includes the stimulation of acute-phase protein release by the liver [19-22], is established and part of this response includes the Systemic Inflammatory Response Syndrome [20]. Most of these changes are signaled by cytokines [20,21]. More specifically, the expression of inducible genes leading to the synthesis of cytokines, chemokines, chemokine receptors, adhesion molecules, enzymes and autacoids relies on transcription factors NF-κB and AP-1, that play a central role in the regulation of these inflammatory mediators [23,24]. The maximum intensity of the immune response may be reached when an associated systemic infection is produced. The excessive consumption of coagulation factors with hyperproduction of anticoagulant factors can induce a state of hypocoagulability or Disseminated Intravascular Coagulation (DIC) that, ultimately, favors bleeding [25] (Figure 1).

During the evolution of the nervous and immune phase of the inflammatory response, the body loses its more specialized functions and structures. In this progressive deconstruction, depletion of the hydrocarbonate, lipid and protein stores occurs [26], as well as multiple or successive dysfunction and posterior failure, apoptosis or necrosis of the specialized epithelium, i.e. the pulmonary, renal, gastrointestinal and hepatic ones [27]. Although these alterations are considered a harmless consequence of the systemic inflammatory response, they are also a mechanism through which there is a redistribution of immediate constituents in the body. In this case, the redistribution of metabolic resources responds to the different trophic requirements of the body as the inflammation progresses [12,14]. It has been proposed that the host is destroying itself [28] which would correspond to autophagy [29-31].

However, consumption of the substrate deposits and the dysfunction or failure of the specialized epithelia of the body could also represent an accelerated process of epithelial dedifferentiation [12,14,32]. The hypothetical ability of the body to involute or dedifferentiate could represent a return to early stages of development. Therefore, it could constitute an effective defense mechanism against injury since it could make retracing a well-known route possible, i.e. the prenatal specialization phase during the last or endocrine phase of the inflammatory response [14]. This specialization would require a return to the prominence of oxidative metabolism, and thus angiogenesis, in the affected epithelial organs to create the capillary bed that would make regeneration of the specialized epithelial cells possible or otherwise to carry out repair through fibrosis or scarring [12,14,15,32].

Thus, the endocrine functional system facilitates the arrival of oxygen transported by red blood cells and capillaries. It is considered that angiogenesis characterizes this last phase of the inflammatory response, so nutrition mediated by the blood capillaries is established. The ability to use oxygen in the oxidative metabolism is recovered when patients recover their capillary function. This type of metabolism is characterized by a large production of ATP (coupled reaction) which is used to drive multiple specialized cellular processes with limited heat generation and which would determine the onset of healing. In the convalescent phase, the dedifferentiated epithelia specialize again, the energy stores that supplied the substrate necessary for this demanding type of metabolism are replete, and complete performance is reached, thus making active life possible [12-14,18] (Figure 1)

Nevertheless, angiogenesis could have other functions in the phases prior of the inflammatory response. The earliness of endothelial proliferation, as well as the ability of these cells to express antioxidant and anti-enzymatic phenotypes [9,11] suggests that early angiogenesis could have a defensive role [18]. If so, in the phases prior to the development of capillaries, the endothelial cells could have the function of reducing oxidative and enzymatic stress that the inflamed tissues and organs suffer.

The expression of the nervous, immune and endocrine functional systems during the inflammatory response, makes it possible to differentiate three successive phases which progress from ischemia, through a metabolism that is characterized by defective oxygen use (reperfusion, oxidative burst and heat hyperproduction or uncoupled reaction) up to an oxidative metabolism (oxidative phosphorylation) with a correct use of oxygen (coupled reaction) that produce usable energy. If so, it is also tempting to speculate on whether the body reproduces the successive stages from which life passes from its origin without oxygen [33] until it develops an effective, although costly, system for the use of oxygen every time we suffer inflammation [12-15,18].

The sequence in the expression of progressively more elaborated and complex nutritional systems could hypothetically be considered the essence of the inflammation, regardless of what is etiology (traumatic, hypovolemic or infectious) or localization may be. Hence, the incidence of harmful influences during their evolution could involve regression to the most primitive trophic stages, in which nutrition by diffusion (nervous system) takes place [12,14]. Thus, the incidence of noxious factors during the evolution of the systemic inflammatory response produces severe hemodynamic alterations again, and lastly, vasodilatory shock with tissue hypoxia and lactic acidosis is established [34]. This mechanism of metabolic regression is simple, and also less costly. It facilitates temporary survival until a more favorable environment makes it possible to initiate more complex nutritional ways to survive (immune and endocrine system) [14,18] (Figure 1).

## Portal hypertension

Portal hypertension (PH) is characterized by an increase in portal vein pressure as a result of the obstruction to portal flow [35,36]. Depending on the level of the obstruction, PH is classified as either prehepatic, intrahepatic or posthepatic [37].

Intrahepatic portal hypertension is most often caused by chronic liver disease, with the majority of preventable cases attributed to excessive alcohol consumption, viral hepatitis, or non alcoholic fatty liver disease [38]. Therefore, in these patients the pathology related to PH is associated to that associated with chronic liver disease. Perhaps this is the reason why the complications suffered by these patients, i.e. hepatorenal syndrome, hepatic encephalopathy, ascites and variceal bleeding, are indistinctly attributed to hepatic disease [38,39] and PH [37].

Prehepatic portal hypertension is most often caused by a cavernoma of the portal vein. This cavernoma is related to acute portal-vein thrombosis and it is developed concomitantly with splenomegaly, portosystemic shunts and the reversal of flow in the unaffected intrahepatic portal veins [40]. It is accepted that these patients have no underlying liver disease and their liver function is expected to remain normal throughout their life [35,40].

Post-hepatic portal hypertension, as the intrahepatic type, is also associated with hepatocellular dysfunction [41]. Therefore, for the experimental study of portal hypertension, the prehepatic type is usually chosen since it has the least degree of hepatic impairment. Particularly, the most frequently used experimental model of prehepatic portal hypertension is that which is achieved by partial portal vein ligation in the rat [42-44].

## Experimental prehepatic portal hypertension

Partial portal vein ligation in various animals, but particularly in the rat, has been widely used for portal hypertension studies [42-45].

The surgical technique most frequently used in the rat was described by Chojkier and Groszmann in 1981 [42]. In brief, the rat is anesthetized and after laparotomy, the portal vein is dissected and isolated. A 20-gauge blunt-tipped needle is placed along-side the portal vein and a ligature (3-0 silk) is tied around the needle and the vein. The needle is immediately removed, yielding a calibrated stenosis of the portal vein.

If it is taken into account that the intensity of the portal hypertension is determined by the resistance to the inflow produced by the constriction of the portal vein conditioning its posterior evolution, this experimental model of prehepatic portal hypertension could be improved by increasing the initial resistance to the blood flow. With this objective in mind, we have modified the surgical technique by increasing the length of the stenosed portal tract with three equidistant stenosing ligations since, according to the Poiseuille equation (R = 8 μL/πr^4^), the resistance (R) to the flow of a vessel depends of the length (L) on the radius (r), and the coefficient of viscosity of the blood (μ). In brief, three partial ligations were performed in the superior, medial and inferior portion of the portal vein, respectively and maintained in position by the previous fixation of the ligatures to a sylastic guide. The stenoses were calibrated by a simultaneous ligation (3-0 silk) around the portal vein and a 20-G needle. The abdominal incision was closed on two layers [46,47].

The mechanisms which contribute to the development and maintenance of portal hypertension change along time in the portal vein ligated (PVL) rat [48,49]. In the first days after portal stenosis, hypertension is attributed to the sharp increase in resistance to the flow caused by the portal stenosis. However, 4 days after portal stenosis, the partial development of portosystemic collaterals reduces the portal venous resistance, and portal hypertension is maintained because of an increased splanchnic venous flow, which is related to intestinal hyperdynamic circulation, established completely at 8 days of evolution [48]. Two weeks after the operation, the animals develop splanchnic and systemic hyperdynamic circulation with derivation of 90% of the portal blood flow through the portosystemic collaterals, which means that there is a decrease in the portal flow that reaches the liver [50,51]. The portal pressure in this evolutive stage is about 15 mmHg, which means an approximate increase of 50% regarding its value in control rats [48].

Portal pressure can be measured by a direct or indirect method. In the first case, it is done by cannulation of the mesenteric vein through the ileocecal vein or a small ileal vein with a PE-50 catheter placing its tip in the distal part of the superior mesenteric vein [52]. The indirect measurement of portal pressure is performed by determining the splenic pulp pressure by intrasplenic puncture inserting a fluid-filled 20-gauge needle into the splenic parenchyma [48]. It has been demonstrated that there is an excellent correlation between splenic pulp pressure and portal pressure [48,50].

**Figure 2:**
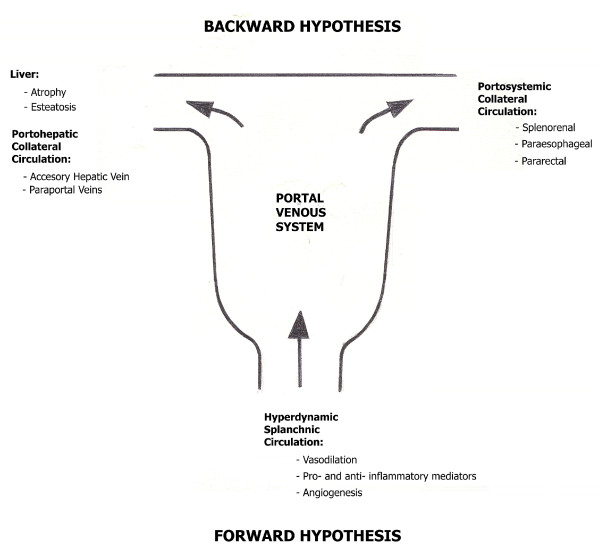
Mechanisms underlying the pathophysiology of short-term prehepatic portal hypertension in the rat.

It has been considered that at two weeks of evolution portal hypertension is a consequence of a pathological increase in the portal venous inflow ("forward" hypothesis) and resistance ("backward" hypothesis) [48,49] (Figure 2). The increase in blood flow in the portal venous system is established through splanchnic arteriolar vasodilation that produces hyperdynamic splanchnic circulation or splanchnic hyperemia [50,51]. In turn, the increase in vascular resistance to the portal blood flow is found in the presinusoidal (partial portal ligation) hepatic circulation, as well as in the portal collateral circulation (enhanced portal collateral resistance) [50,51,53]. Therefore, it is accepted that normalization of elevated portal pressure can only be achieved by attempting to correct both, elevated portal blood flow and elevated portal resistance [52]. However, the splanchnic lymphatic flow could influence the intensity of portal hypertension. Indeed, the gastrointestinal tract could become edematous in portal hypertension, and associated with lymph vessels dilation [54]. It is possible that dilation of lymph vessels is related to the absorption of excess interstitial fluid, resulting from congestion [54]. Therefore, the interstitial edema and the ability to be drained by the lymph vessels could constitute conditioning factors of the intensity of portal hypertension. Thus, the increased splanchnic lymphatic flow would reduce the interstitial edema and would favor the blood flow through the portal venous system.

Hyperdynamic circulation in short-term PVL rats has been principally attributed to two mechanisms: Increased circulating vasodilators and decreased response to vasoconstrictors [53,55], like nitric oxide (NO), carbon monoxide (CO), alpha tumoral necrosis factor (TNF-α), glucagon, prostacycline (PGI_2_), endothelium-derived hyperpolarizing factor, endocannabinoids, adrenomedullin and hydrogen sulfide (H_2_S) [56]. In turn, the hyperactivity to the vasoconstrictors, that is, to endogenous (norepinephrine, endothelin, vasopressin) or exogenous (alpha agonists) ones reflect the impaired vasoconstrictor response, which contributes to vasodilation [57]. Furthermore, it is conceivable that there might be different mechanisms underlying the hypereactivity to vasoconstrictors in portal hypertension.

**Figure 3:**
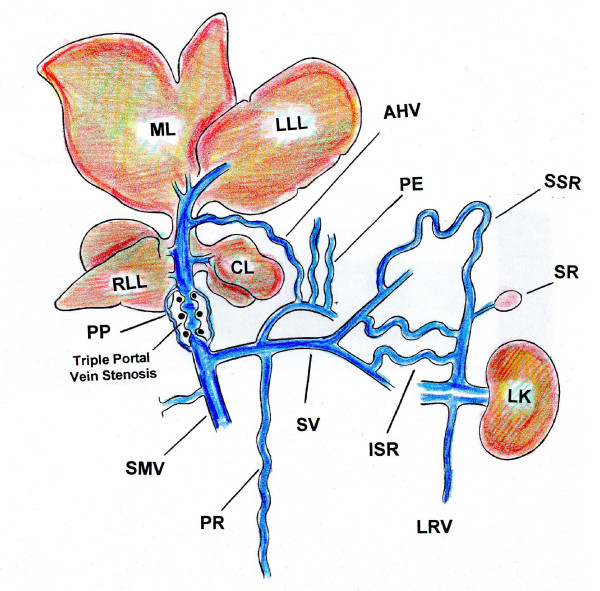
Types of portosystemic collateral circulation in rats with partial portal vein ligation. ML: middle lobe; LLL: left lateral lobe; RLL: right lateral lobe; CL: caudate lobe; AHV = Accesory Hepatic Vein; PP: paraportal; SMV: superior mesenteric vein; PR: pararectal; SV: splenic vein; ISR: inferior splenorenal; SSR: superior splenorenal; PE: paraesophageal; LK: left kidney; SR: suprarenal gland; LRV: left renal vein.

In this evolutive phase of prehepatic portal hypertension in the rat, mainly two types of portosystemic collateral circulation are established: splenorenal and paraesophageal [58]. The development of the portal collateral venous system is not only due to the opening of preexisting vessels, but also to new vessel formation, which is a very active process. Particularly, it has been shown that portal hypertension in the rat is associated with vascular endothelial growth factor (VEGF) induced angiogenesis [59] (Figure 3).

It is considered that portal vein stenosis does not produce liver damage [43]. However, partial portal vein ligation in the rat produces hepatic atrophy with loss of the hepatic sinusoidal bed and it is the cause of elevated resistance to portal blood-flow [52]. However, the degree of hepatic atrophy at 6 weeks post-stenosis of the portal vein is not homogenous and there are some cases in which the hepatic weight increases in regards to the control rats [58]. The different evolution in hepatic weight in the rats with prehepatic portal hypertension is an interesting finding since it demonstrates the existence of a heterogeneous hepatic response in this experimental model.

## Evolutive phases of experimental prehepatic portal hypertension and the splanchnic inflammatory response

It has been suggested that the rat model of gradual portal vein stenosis is much more homogenous than human portal vein obstruction, because it has a narrow range of portal hypertension, degree of portosystemic shunts and hepatic atrophy [60]. However, PVL rats are far from having a uniform evolution, since they can present a wide variability in both hepatic weight (degree of liver atrophy) [58] as well as in the type and degree of portosystemic collateral circulation developed [49,58]. Furthermore, the variability of this experimental model of prehepatic portal hypertension is not only observed in short-term evolution (14 to 28 days) which is where it is studied most, but also in chronic evolutive stages (6 to 14 months) [61].

All of the variations presented by the animals after PVL, aside from invalidating the experimental model and thus disappointing the investigator, probably add complexity and even more importantly, pose problems that are tempting challenges for the investigator. It is also possible that the knowledge of the etiopathogenic mechanisms involved in the evolutive variability of this experimental model will make it easier to understand the evolutive characteristics of human portal hypertension [62].

The different mechanisms that contribute to the development of prehepatic portal hypertension in the rat make it possible to attribute different evolutive phases to this disease [48,49]. The study of the late evolutive phases could be considered of greater interest since the mechanisms involved in its production as well as the disorders that it causes, would be more similar to those that have been described in the human clinical features, since they are related to the chronicity of portal hypertension, among other factors [61].

One of the reasons that this prehepatic portal hypertension experimental model presents great evolutive variability could be based on its inflammatory nature. If so, it would be the individual variability of the inflammatory response intensity, inherent to portal hypertension, which would condition the different evolution in the animals. In this way, the pathogenic mechanisms proposed for the post-traumatic inflammatory response as phylogeny unifiers, and therefore for the category of generics [15], could also participate in the production of the alterations associated with portal hypertension.

Portal hypertension is essentially a type of vascular pathology resulting from the chronic action of mechanical energy on splanchnic venous circulation. This kind of energy can stimulate the endothelium which, owing to its strategic position, plays an exceedingly important role in regulating the vascular system by integrating diverse mechanical and biochemical signals and by responding to them through the release of vasoactive substances, cytokines, growth factors and hormones [9-11]. Mechanical energy may also act in the vascular endothelium as a stress stimuli, generating a inflammatory response [63]. If it is considered, in the case of portal hypertension, that there is an endothelial inflammatory response induced by mechanical energy that affects the splanchnic venous circulation and, by extension, the organs into which its blood drains, it could be speculated that there is a common etiopathogeny that integrates the pathophysiological alterations presented by these organs [18,62].

Several of the early as well as the late morphological and functional disorders presented by the splanchnic organs in experimental prehepatic portal hypertension make it possible to suspect that inflammatory type mechanisms participate in their etiopathogeny [5,6,18,62].

The evolution of portal hypertension as an inflammatory response would be comprised of three phenotypes with a trophic meaning, as previously proposed for the post-traumatic inflammatory response [12-14]. In this response, the ischemia-reperfusion phenotype (nervous functional system) causes edema and oxidative and nitrosative phenotype (immune functional system), inflammatory cells and bacteria are involved in the metabolic activity through the development of enzymatic stress. Lastly, the angiogenic phenotype (endocrine functional system) would be predominated by angiogenesis and its objective is tissue repair [5,6,18,62].

Enteropathy and encephalopathy are between the most important splanchnic and systemic manifestations derived from experimental portal hypertension. In both anatomical sites, gastrointestinal tract and liver, inflammatory pathophysiological mechanisms come together to produce complications characteristic of the PVL rats [18].

## Portal hypertensive enteropathy

The gastrointestinal tract immediately and directly suffers the sudden increase in venous pressure produced by the PVL. In an early evolutive period, portal venous hyperpressure is highest [48,49] when portosystemic collateral circulation has not yet developed, and the mucosa ischemia is an immediate consequence of intestinal venous stasis. The increase in mesenteric venous pressure alters the distribution of blood flow within the bowel wall, decreasing mucosal blood flow and increasing muscularis blood flow. Mucosal hypoxia is related to the constriction of mucosal arterioles, meanwhile the dilation of arterioles in the muscularis increases the blood flow in this layer [64].

Ischemia/reperfusion injury is an important mechanism of mucosal injury in acute and chronic intestinal ischemic disorders [65]. Hypoxia in the intestinal mucosa causes oxidative and nitrosative stress, but also through hypoxia inducible factor-1 (HIF-1), it enhances the expression of hypoxia responsive genes, and therefore improves cell survival in conditions of limited oxygen availability [63].

Two days after PVL in the rat, portal hyperpressure is associated with intraperitoneal free exudates, peripancreatic edema, hypoproteinemia and hypoalbuminemia. The inflammatory nature of these alterations can be hypothesized, since the oral administration of budesonide prevents these early exudative changes [66]. The acute inflammatory endothelial response can cause exudation related to an endothelial permeability increase, which is the cause of swelling and production of peritoneal exudates in this early evolutive phase of portal hypertension in the rat [66]. The inhibition of this inflammatory response by budesonide would indicate the efficacy of this steroid in the prophylaxis of this early acute response. It could be speculated that budesonide produces a down-regulation of the pro-inflammatory mediators partially due at least to an inhibitory effect on the transcription factors that regulates inflammatory gene including AP-1 and NF-κB, that is, through mechanisms similar to those that also act with great efficiency on the allergic inflammatory response to allergens [67,68].

And so we have shown that prophylaxis with Ketotifen, an anti-inflammatory drug that stabilizes mast cells [69], reduces portal pressure, the number of degranulated mast cells in the cecum and the concentration of rat mast cell protease II (RMCP-II) in the mesenteric lymphatic nodes of rats with early prehepatic portal hypertension [70]. Histamine and serotonin stand out among mediators released by mast cells and cause vasodilation and edema due to increased vascular permeability [71]. Neutral proteases may also regulate the tone of the splanchnic vascular bed and provoke edema and matrix degradation. Particularly RMCP-II, considered a specific marker of rat mucosal mast cell degranulation, can modulate the vascular function through their ability to convert Angiotensin I to Angiotensin II. It also may promote epithelial permeability. Angiotensin II is a powerful vasoconstrictor that produces mucosal ischemia and also increases vascular permeability and promotes recruitment of inflammatory cells into tissues [71]. Furthermore, both Angiotensin II, which produces vasoconstriction and mucosal ischemia, and RMCP-II, which increases intestinal permeability and enhanced antigen and bacteria uptake, consequently induced bacterial translocation to the mesenteric lymph nodes where they would activate a "chemotactic call" to mast cells and worsen inflammatory responses [71,72]. Therefore, Ketotifen could inhibit mast cell migration and activation in the mesenteric lymph nodes and thus reduce the release of mediators involved in the development of the increased portal venous inflow that causes portal hypertension in short-term PVL rats [70].

The intestinal effects of portal hypertension are not only harmful, since in this case the sudden obstruction of the portal venous flow would possibly cause death, which normally does not occur [61,62]. So, in this early evolutive phase, rats have reduced serum concentrations of mediators considered pro-inflammatory, as are PGE_2 _and LTC_4 _[73]. The migration of mast cells from the intestinal mucosa to the lymph nodes can also be beneficial in order to avoid the development of an "inflammatory battle" mediated by mast cells in the intestinal mucosal layer [18,73].

In a later evolutive phase (4 days) portal hypertension is associated with features of hyperdynamic circulation. In the first 24 hours after the operation, hypoxia in the mucosa may stimulate the upregulation of e-NOS in the intestinal microcirculation with NO hyperproduction. This increase in eNOS expression occurs through VEGF upregulation and subsequent AKT/proteinkinase B activation in highly vascularized areas of the mucosa, and might initiate the cascade of events leading to hyperdynamic splanchnic circulation in prehepatic portal hypertension [74,75]. Therefore, the development of hyperdynamic circulation occurs gradually from the initial stages of prehepatic portal hypertension in the rat and is associated with the development of portosystemic shunting [74].

**Figure 4:**
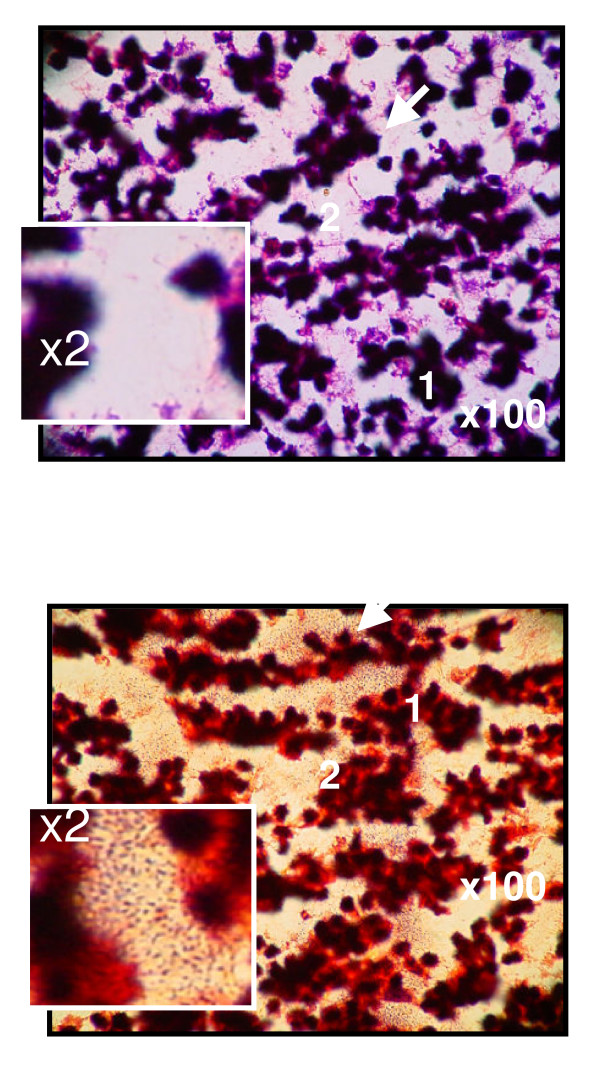
Microscopic images from mesenteric lymph node (1) corresponding to: A. Control; B: Portal-hypertensive rats at 1 month of evolution. In portal hypertensive-rats microorganisms infiltrate significantly the lymph nodes (arrows). Gram stain ×100.

In prehepatic portal hypertension in the rat, bacterial translocation is an early event. Two days after the PVL, it has been demonstrated that a significant greater portion of rats had positive mesenteric lymph node cultures [76] (Figure 4) and coincides with the establishment of hyperdynamic and portosystemic splanchnic circulation [18]. Bacterial translocation to the superior mesenteric lymph nodes is attributed to a bacterial overgrowth, disruption of the gut mucosal barrier and impaired host defenses [77-79]. In portal hypertensive rats related to other models of portal hypertension, like CCL_4_, CBDL or TAA, the event of bacterial translocation is also produced.

A microscopic splanchnic alteration that is usually present in stenosed portal vein ligated rats is dilation and tortuosity of the branches of the upper mesenteric vein. We have called this alteration "mesenteric venous vasculopathy" [61]. In early stages, four weeks postoperatory, mesenteric venous vasculopathy could be attributed to the hyperdynamic splanchnic circulation [62].

**Figure 5:**
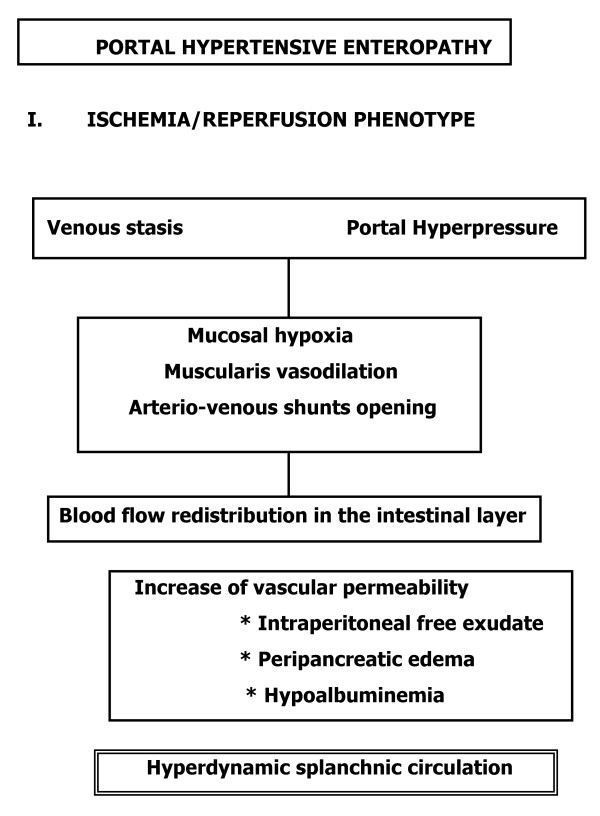
Etiopathogenic mechanisms in the successive phases of the hypertensive portal enteropathy in the rat. Ischemia/Reperfusion phenotype.

**Figure 6:**
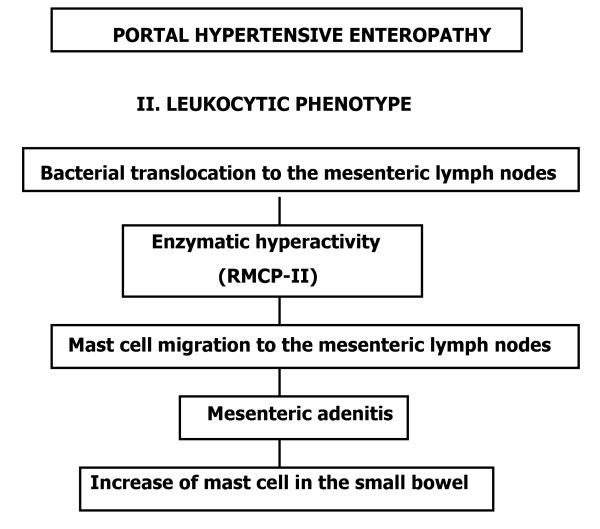
Etiopathogenic mechanisms in the successive phases of the hypertensive portal enteropathy in the rat. Leukocytic phenotype.

**Figure 7:**
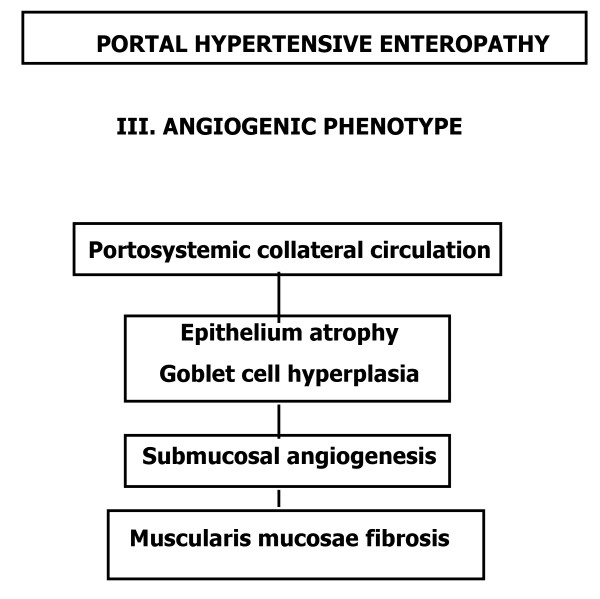
Etiopathogenic mechanisms in the successive phases of the hypertensive portal enteropathy in the rat. Angiogenic phenotype.

Since 1985, when McCormack et al. [80] described hypertensive gastropathy in patients with portal hypertension, successive histological studies on the remaining portions of the gastrointestinal tract have demonstrated that alterations similar to gastric ones are found in the duodenum, jejunum, ileum, colon and rectum [81,82]. Since the basic structural alteration found in the gastrointestinal tract is vascular and consists of increased size and number of the vessels, the very appropriate name of "hypertensive portal intestinal vasculopathy" has been proposed [83]. However, in addition to vascular alterations, histological evidence of non-specific inflammation has been described in the gastropathy, enteropathy and colopathy associated with portal hypertension [80-82]. The chronic inflammatory infiltration found in the small bowel predominantly consists of mononuclear cells and it is associated with atrophy, a decreased villous/crypt ratio, edema of the lamina propria/bowel wall, fibromuscular proliferation and thickened muscularis mucosa [81,84]. Because most of the aforementioned characteristics can be explained on the basis of increased levels of mast cell mediators [71], these cells could be involved in the pathogenesis of portal hypertensive enteropathy [5] (Figures 5, 6 and 7).

Portal hypertensive rats at six weeks of evolution show increased mast cell infiltration in the duodenum, jejunum, ileum and superior mesenteric lymph node complex [85,86]. Mast cells are selectively found in relatively large numbers adjacent to blood or lymphatic vessels but are most prominent immediately beneath the epithelial surface of the skin and in the mucosa of the genitourinary, respiratory and gastrointestinal tracts, the latter having greater density. This selective accumulation at tissue sites where foreign materials attempt to invade the host suggests that mast cells are among the first cells to initiate defense mechanisms [87]. This function of mast cells, especially in the gastrointestinal tract, which provides a barrier against infection, could explain their increase in the small bowel in rats with prehepatic portal hypertension [86]. Mast cells have the unique capacity to store presynthesized TNF-α and thus can release this cytokine spontaneously after their activation [88]. Therefore, the excess number of mast cells in the small bowel and in the mesenteric lymph node complex of rats with portal hypertension could be related to their ability to release the stored TNF-α when the appropriate stimulus is acting. It has been hypothesized that TNF-α causes vasodilation through both the prostaglandin and nitric oxide pathways [88]. If so, the release of the stored TNF-α by activated mast cells may be involved in the development of the hyperdynamic circulatory syndrome [89]. To be specific, hyperdynamic splanchnic circulation that increases portal venous inflow would help to maintain long-term portal hypertension which in turn produces dilation and tortuosity of the branches of the upper mesenteric vein, that is, mesenteric venous vasculopathy [82].

The activation of the mast cells in the mesenteric lymph nodes in rats with portal hypertension, would not only collaborate in the production of mesenteric adenitis, but also would constitute a source of mediators for the inflammatory response between the intestine and systemic blood circulation [86]. The lymph tissue associated with the intestine constitutes the largest lymphatic organ of the body and its activation in portal hypertensive enteropathy would produce the release of inflammatory mediators. These would be transported by the intestinal lymph vessels to the pulmonary circulation -inducing an inflammatory phenotype- and later to the systemic circulation. The priority of mesenteric lymph node circulation with respect to portal circulation for transporting pro-inflammatory mediators released in the intestinal wall in different pathologies related to intestinal ischemia, such as hemorrhagic shock or serious burns [90], suggests that in other pathologies that also produce intestinal ischemia, like prehepatic portal hypertension, the mesenteric lymph is a regional pro-inflammatory mediator vehicle, that is, a splanchnic one, but with a systemic effect [62] (Figure 6).

The ability of the mast cells for the synthesis and selective or dedifferentiated release of different mediator molecules of the inflammatory response would explain their participation in multiple and different pathological processes, as well as in the different evolutive phases of prehepatic portal hypertension. With respect to the splanchnic inflammatory response induced by portal hypertension, the mast cells could participate in the initial or acute phases, producing vasodilation, increased endothelial and epithelial permeability, edema, increased lymphatic flow and mesenteric adenitis, as in the more advanced, late or chronic phases. In the last phases, the chemotactic factors derived from the mast cells stimulate the proliferation of fibroblasts and the synthesis of collagen. Meanwhile, histamine and heparine promote the formation of new blood vessels. Both fibrogenesis and angiogenesis are responsible for fibromuscular and vascular proliferation in the intestinal wall, respectively [62].

In portal hypertensive rats six weeks after the operation, the increase in diameter and number of blood vessels in the submucosa has already been shown in the duodenum, which at the same time is correlated with the infiltration by the mast cells [85]. Therefore, vasodilation and angiogenesis which are responsible for the increase in size and number of vessels, and in turn, for vascular structural alterations that characterizes portal hypertensive enteropathy [81,83] can be attributed to, among other factors, the pathophysiological effects produced by the excessive release of mast cell mediators [85,86] (Figure 7).

Splanchnic hyperemia, increased splanchnic vascularization and the development of portal-systemic collateral circulation in portal hypertensive rats are partly a VEGF-dependent angiogenic processes [59,91]. This angiogenic hyperactivity that occurs in the prehepatic portal hypertensive model could be mediated by mast cells [85,86].

There are multiple factors involved in the development and enlargement of portosystemic collaterals, which regulate the collateral flow [5]. At two weeks of the postoperatory period, portal hypertensive rats develop splanchnic hyperdynamic circulation with a derivation of 90% of the portal blood flow through the portosystemic collaterals [50]. Extrahepatic portosystemic collateral circulation persists in the long-term [3, 6 and 12 months] [47,58]. However, in these chronic evolutive phases, although the animals present collateral circulation, this is not always associated with portal hypertension [61,62]. It has been proposed that long-term vasculopathy in portal hypertensive rats constitutes a remodeling process not associated with portal hypertension [92].

The structural changes that are produced in the long-term in prehepatic portal hypertension in the rat could be similar to those described in other chronic inflammatory processes. These morphological alterations would not only be vascular, both macro- and microscopic, but also the rest of the intestinal structures would participate in greater or lesser intensity [93]. In particular, the morphological vascular alterations stand out in chronic portal hypertensive enteropathy. However, we have also described epithelial remodeling, which consists in goblet cell hyperplasia [94]. Goblet cell hyperplasia with mucus hypersecretion is an alteration characteristic of epithelial remodeling of the respiratory tract in chronic inflammatory processes, as are asthma and chronic obstructive pulmonary disease [95-97]. And so, goblet cell hyperplasia could be attributed to chronic hypertensive portal enteropathy in the rat. [94].

## Steatosis related to portal hypertension

One of the reasons why the prehepatic portal hypertension experimental model in the rat is far from having a uniform evolution, is because it presents a wide variability in hepatic weight [78,81].

The wide variation of hepatic weight presented by the portal vein ligated rats in both early as well as late evolutive phases suggests that the liver could be one of the factors that determine the evolutive heterogeneity of this experimental model [58]. If the animals are distributed according to their hepatic weight in each evolutive phase, from more to less, in three groups called A, B and C, a cluster analysis shows that in early evolutive phases (6 weeks) of experimental prehepatic portal hypertension, the percentage of animals with less hepatic weight is greater (group C). On the contrary, in the late evolutive phases (6, 12 and 14 months) the percentage of animals with greater hepatic weight (group A) increases progressively [61]. Thus, it could be considered that the hepatic atrophy (group C) that characterizes the early evolutive stages of prehepatic portal hypertension in the rat may be a reversible alteration in the long-term. It is significant that the animals belonging to group A, although they are characterized by the increase in hepatic weight, also present portosystemic collateral circulation [58,61].

**Figure 8:**
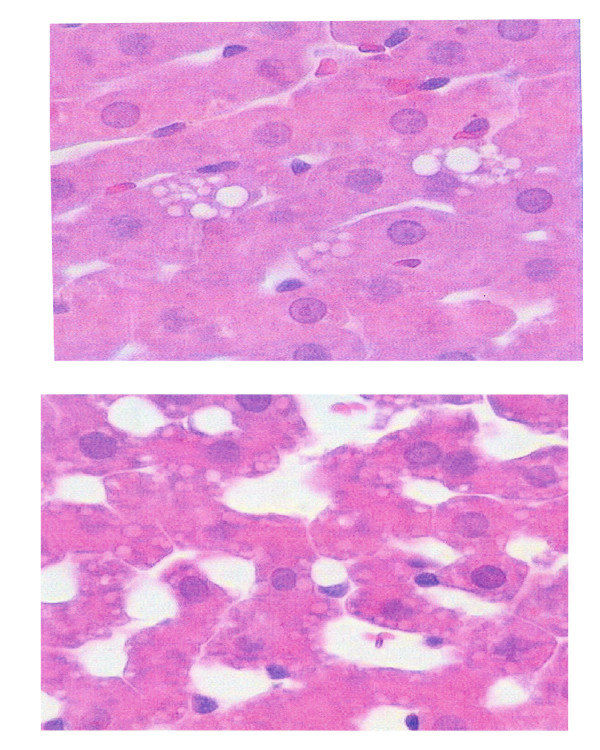
Liver steatosis in experimental prehepatic portal hypertension (superior: 1 month after the operation; inferior: 1 year after the operation; H&E; ×40).

A histological study of the liver, performed in order to verify if the existence of a liver pathology could justify this wide spectrum of liver weight, has demonstrated that hepatocytic fatty infiltration exists in portal prehepatic hypertensive rats [98]. It has also been verified in this study that the fat accumulation in the hepatocytes progressives from a short- (1 month) to a long-term (1 year) evolutive stage of portal hypertension, and thus the persistence of etiopathogenic mechanisms involved in its production could be considered [98]. Liver steatosis could also be the cause of the hepatomegaly which characterizes portal prehepatic hypertensive rats belonging to group A. If so, it could be considered that partial portal ligation not only makes it possible to obtain an experimental model of portal hypertension but also a steatosis model (Figure 8).

Hepatic steatosis alone is thought to be the most common form of nonalcoholic fatty liver disease (NAFLD) and is considered "benign", but not quiescent. In this way, the NAFLD spectrum is wide and ranges from simple fat accumulation in hepatocytes (fatty liver), without biochemical or histological evidence of inflammation or fibrosis, to fat accumulation plus necroinflammatory activity with or without fibrosis (steatohepatitis) to the development of advanced liver fibrosis or cirrhosis (cirrhotic stage) [99,100]. However, although a progressive hepatocytic fatty infiltration during their chronic evolution is produced in partial portal vein ligated rats, this is not associated with histological signs of inflammation or fibrosis. The hepatic steatosis could therefore be considered a "benign" type of the larger spectrum of NAFLD in these rats with prehepatic portal hypertension [98].

The mechanisms by which portal hypertension could induce liver steatosis are not fully understood. In prehepatic portal hypertensive rats at 6 weeks of evolution, the increase of TNF-α, IL1β and NO in the liver is associated with megamitochondria [101]. The reduced portal flow produced related to the portal stenosis could be involved in megamitochondria formation because hypoxia and anoxia are known to induce magamitochondria [102] and the mitochondrial function is impaired early by the extrahepatic portal obstruction in the rat [103]. Also, TNF-α and TNF-related cytokines can contribute to the liver steatosis because they stimulate hepatic lipogenesis and increase the plasma levels of free fatty acids and triglycerides [104]. Mitochondrial alterations are also produced by NO. The increased synthesis of NO associated with reactive oxygen species (O_2 _^-^) induces peroxynitrite (ONOO^-^) formation, which in turn inhibits various mitochondrial respiratory chain complexes [105].

Possible factors involved in fat accumulation in the hepatocytes also include components of the neuroendocrine response to portal hypertensive stress, among others. Specifically, corticosterone and glucagon, which increase in this experimental model, promote lipolysis in fat tissue and a plasma increase of free fatty acids. Therefore, both hormones could produce an excess "input" of fatty acids to the liver [101]. Insulin resistance is the most constant pathogenic factor in patients with a liver disease by fat storage [106,107]. In portal hypertension, this resistance can be induced by both glucocorticoids and TNF-α. Both mediators would contribute to hepatic steatosis by this mechanism because they would favor peripheral lipolysis and the uptake and mass deposition of free fatty acids in the liver [101].

Prehepatic portal hypertension in the rats, both in the short- (1 month) and in the long-term (1 year) produce hepatic accumulation of triglycerides and cholesterol [108]. In the long-term (2 years), the plasmatic increase of low density lipoprotein (LDL) and lipopolysaccharide binding protein (LBP) is associated with the reduction of high-density lipoproteins (HDL) and triglycerides. The increased influx of free fatty acids beyond the metabolic requirements leads to their storage as triglycerides, which results in steatosis and provides substrate for lipid peroxidation [109]. Since the accumulation of triglycerides and cholesterol in the hepatocytes persisted in the long-term evolutive stage of prehepatic portal hypertension, possibly, the etiopathogenic mechanisms involved in its production could also persist [108]. This persistence in the alterations of lipid metabolism has characteristics that could be related to the existence of a chronic inflammatory hepatic state [100]. The association of fatty liver and liver inflammation supports the etiopathogenis of other diseases, such as type II diabetes, dyslipidemias, obesity and metabolic syndrome [109]. In particular, the metabolic syndrome consists of a cluster of metabolic conditions, such as hyper-LDL, hypo-HDL, insulin resistance, abnormal glucose tolerance and hypertension [110]. Interestingly enough, most of these metabolic conditions have also been described in prehepatic portal hypertensive rats.

Furthermore, the mechanisms that have been proposed in order to explain the pathogeny of the fatty liver disease also correspond with those expressed for the inflammatory response [12-15]. The excess cellular oxidative and nitrosative stress, mediated by ROS/RNS [110], the hyperactivity of inflammatory cells in the liver, such as Kupffer cells [111] and mast cells [112] and pro-inflammatory cytokines stand out [113]. As a result, it could be considered that in prehepatic portal hypertension, as in obesity and in the metabolic syndrome, the NAFLD represent the result of a low-grade chronic inflammatory state [100,113]. The establishment of a fatty liver could have a similar meaning to what is proposed for the inflammatory response. This would mean a regression to the periods of evolution with metabolic characteristics that are similar to those imposed by steatosis.

From an embryological point of view, the liver can be thought of as a substitute of the yolk sac. In all vertebrates, the liver develops in close association with the yolk sac [114,115]; in cyclostomata and amphibia it develops directly from it. In mammals the liver develops in close association with the non-functional yolk sac, the placenta temporarily takes the place of the intestine and the umbilical vein assumes the role of the portal vein for some time [114]. A major function of the yolk sac is associated with the accumulation of fat [116]. The yolk sac plays a vital role in providing lipids and lipid-soluble nutrients to embryos during early phases of development [116,117]. Particularly, the yolk sac uses HDL and VLDL as carriers to incorporate cholesterol from the maternal circulation and to transfer it to the embryonic side [116]. In experimental prehepatic portal hypertension, the liver could constitute as a kind of yolk sac in which the animal carries out a pathological deposit of lipids. In this hypothetical situation, through the expression of inflammatory mediators, the liver would be able to regress to evolutive phases in which the metabolic characteristics were suitable.

It has been proposed that the failure to upregulate fatty acid oxidation systems and the ensuing burning of energy in the liver may play a role in the modulation of hepatic steatosis [118]. The liver could respond to portal hypertensive stress with a transcriptional response that causes a shift or transition to lipid metabolism by reducing burned energy which leads to lipid storage [118]. In poikilothermic animals, with large fluctuations in their core temperature, transcript profiles of liver also showed cold-induced transitions to lipid metabolism [119]. Poikilotherms also stored lipids in several storage organs, including the liver [120]. Perhaps, by remembering the old poikilothermic metabolism, through reorganization the lipid metabolism, the liver would develop a metabolic strategy in portal hypertension.

## Extra-splanchnic alterations in portal hypertension

Extra-splanchnic alterations are circumstantial in prehepatic portal hypertension and constitute the clearest argument in favor of its systemic nature.

### * Portal hypertensive encephalopathy

Prehepatic portal hypertension in humans is associated with neuropsychological and brain magnetic resonance changes consistent with minimal hepatic encephalopathy [121]. Since intrinsic hepatocellular disease does not exist in this type of portal hypertension, the existence of a portal-systemic bypass is the principal cause of minimal hepatic encephalopathy. Consequently, this hepatic encephalopathy is categorized as type B [122].

The partial portal vein ligated rat model could be appropriate for the experimental study of the minimal hepatic encephalopathy related to prehepatic portal hypertension because portal-systemic shunting is developed. Hence, it should be considered that an associated hepatic pathology exists [98].

The important role that inflammation has on the modulation of the molecular pathogenesis of hepatic encephalopathy has recently been highlighted [123,124]. Inflammation, however, may not only be limited to modulating the severity of hepatic encephalopathy but also could indeed be its own pathophysiological mechanism [125]. If so, inflammation of the central nervous system, when related to prehepatic portal hypertension, could be the basic mechanism that drives the essential nature of minimal hepatic encephalopathy.

At one month of evolution, prehepatic portal hypertensive rats present increased SDF-1 alpha levels in the hippocampus and cerebellum associated with increased TNF-α and CXCR4 levels in the hippocampus and decreased RANTES levels in the striatum [126]. The increase of the chemokine system CXCR4/SDF-1 alpha in the hippocampus could be related to a remodeling structural process since SDF-1 alpha is a pro-inflammatory cytokine that regulates neurodevelopmental processes in the central nervous system as well as neuronal migration [127]. Furthermore, the increase of SDF-1 alpha in the cerebellum could regulate the neuronal rearrangement or neurogenesis [126].

Chemokines have a dual role as neurodegenerative or neuroprotective molecules in the central nervous system. In experimental portal hypertensive encephalopathy, chemokines can contribute to creating an immune phase in the hippocampus and cerebellum that does not necessarily involve just harmful phenomena, but rather exerts a beneficial remodeling effect. The objective would be to adapt cerebral areas to the new metabolic state created by portal hypertension [125]. At the same time, the brain changes demonstrated in this experimental model of portal hypertension could be related to the development of a minimal hepatic encephalopathy [126].

It is now generally accepted that mast cells are present in the normal brain in many mammalian species, including humans and rodents. Since these cells, when activated, could translocate from the splanchnic area to the central nervous system [128] we have hypothesized that mast cells would be involved in a splanchnic-brain chemokine-mediated crosstalk [126].

Other alterations that have been described in this experimental model could also be related to the establishment of a low grade cerebral inflammatory response. These include, for example, an altered blood-brain barrier permeability [129], neuro-endocrine alterations [46,130,131] with a decreased uptake and an increased release of norepinephrine [130], an upregulation of tyrosin hydroxilase activity [132], as well as astrogliosis and angiogenesis in the hippocampus [133]. These functional, biochemical and morphological alterations may possibly help characterize portal hypertensive encephalopathy. In the early evolutive phases, portal hypertension and portosystemic collateral circulation are important pathogenic factors for the production of the encephalopathy. However, in later phases, both factors lose their initial leading role, as the progression of hepatic steatosis is more and more influential [134].

Cardiovascular and metabolic derangements in prehepatic portal hypertensive rats are related to pathologic changes in regulatory mechanisms in the central nervous system. Central deregulation, i.e. brain stem cardiovascular nuclei, contributes to blunted cardiovascular responsiveness in prehepatic portal hypertension [135]. Also the anomalous metabolic response, characterized by steatosis [98] can be attributed to altered homeostatic responses by the brain-splanchnic axis [136-139].

### * Hepatopulmonary syndrome

Two pulmonary vascular disorders can occur in liver disease and/or portal hypertension: the hepatopulmonary syndrome, which is characterized by intrapulmonary vascular dilations, and portopulmonary hypertension, in which pulmonary vascular resistance is elevated [140]. The exact pathophysiological mechanisms of these pulmonary vascular disorders are unknown. However, as hepatopulmonary syndrome and portopulmonary hypertension have been reported in patients with extrahepatic portal hypertension, the common factor that determines their development must be portal hypertension [140,141].

It is accepted that partial portal vein ligation in the rat does not result in the development of hepatopulmonary syndrome [142]. However, exogenous administration of endothelin-1 to partial portal vein ligated rats increased TNF-α levels, increased pulmonary cNOS production and pulmonary intravascular macrophage accumulation, and led to the development of hepatopulmonary syndrome. These findings support an important role for increased circulating endothelin-1 in the development of experimental hepatopulmonary syndrome and suggest that endothelin-1 and TNF-α have synergistic effects on the pulmonary microvasculature in portal hypertension [143]. Taking into account that these results have been obtained from early stages of the hepatopulmonary syndrome, perhaps there are other factors that condition its evolution in the long-term.

The hepatopulmonary syndrome is a consequence of abnormal angiogenesis of the pulmonary microcirculation induced by portal hypertension [140]. Therefore, a remodeling process is produced. Pulmonary remodeling involves distal vessels and the vascular abnormalities include increased numbers of dilated precapillary and capillary vessels and precapillary arteriovenous communications [144]. Thus, the study of the implications of abnormal angiogenesis in the pulmonary circulation of long-term portal hypertension in rats, would contribute very interesting information for evaluating this complication in the experimental model.

### * Portal hypertensive kidney

Sodium retention along with peripheral vasodilation are features of prehepatic portal hypertension. However, in portal vein ligated rats, sodium retention occurs only when a factor that produces decompensation is involved, for example, a liver function-dependent factor [145].

The existence of peripheral vasodilation is an important predisposing factor for developing prerenal failure in rats with prehepatic portal hypertension. A factor that causes extreme underfilling of the arterial circulation and therefore renal hypoperfusion in this experimental model would favor the production of acute renal failure (preischemic state) [146]. If so, the hemodynamic alterations affecting the kidney parenchyma associated with sodium retention could represent a functional impairment similar to that which affects other organs in Multiorgan Dysfunction Syndrome (MODS).

## Portal hypertensive metabolic syndrome

In rats with prehepatic portal hypertension, the sum of the splanchnic (hepato-intestinal) and extra-spanchnic (systemic) alterations allows for proposing a hypothetical portal hypertensive syndrome. During the evolution of this syndrome, the hemodynamic changes that play the leading roles in the early evolutive phases are replaced later by the metabolic alterations.

Hyperdynamic splanchnic and systemic circulation are early hemodynamic alterations in this experimental model, and are associated with the development of portosystemic collateral circulation [48-50]. Hyperdynamic circulation can achieve two objectives: the first, the modulation of relative hypoxia that the tissues can suffer when the blood flow is increased, thus reducing the time needed for extracting oxygen. And second, the production of a "splanchnic steal" phenomenon, progressive and unyielding vasodilation [147] that leads to sodium and water retention and increased blood volume. The body essentially becomes salinized and hydrated.

Both objectives of hyperdynamic circulation could be considered the result of an ischemia-revascularization phenomenon, but a "masked" one since it essentially would produce oxidative and nitrosative stress related to the relative tissue hypoxia, and consequently hydration or swelling [13,14]. Since the ischemia-revascularization phenomenon has been considered the initial phase of the systemic inflammatory response in serious injuries [13-15], the pathogenic mechanisms involved in the splanchnic and systemic hyperdynamic circulation could represent triggering mechanisms of the systemic inflammatory response, whether low or high grade, in experimental prehepatic portal hypertension [62].

This systemic inflammatory response progresses through the induction by oxidative stress to an acute response phase. Since in these initial phases of prehepatic portal hypertension, there is no significant degree of hepatic or intestinal failure, both organs are capable of carrying out an acute phase response that offers the suitable mediators for continuing the inflammatory response already underway and for regulating the enzymatic tissue stress associated with this phase [62,148]. The hyperproduction of chemokines, cytokines, cytokine receptors and adhesion molecules in this phase, should also be modulated by the acute phase splanchnic response [148-150]. The persistence of oxidative and enzymatic stress makes the inflammatory response chronic.

The chronicity of such inflammatory response is perhaps the fundamental factor so that more metabolic alterations progressively develop. And as a result, the body adapts to the new situation or state created by portal hypertension. Thus, in rats with prehepatic portal hypertension, tissue remodeling processes are established in the long-term by angiogenesis and fibrogenesis [93]. One of the most important metabolic changes is hepatic steatosis [98,101,108]. The impairment of the lipid metabolism in this experimental model of portal hypertension confirms the name that has been proposed for this system, since it has certain similarities to the Metabolic Syndrome [108,109]. In this sense, prehepatic portal hypertension, in addition to the alterations of inflammatory nature produced in the hippocampus and cerebellum [126], is associated with the impairment of spatial reference memory [151]. All these alterations that have been described in the Central Nervous System of this experimental model [126,129,130,151] suggest that there is subclinical or minimal encephalopathy [151]. The alterations in attention and memory that characterize this kind of encephalopathy have also been described in human depression, a physical and psychological disorder that affects every aspect of human physiology [109]. The exact relationships between lipid metabolism and immune abnormalities in depression are still unknown [109,152] although it has been suggested that patients with NAFLD and patients suffering a depression are characterized by a low-grade systemic inflammation [153].

Furthermore, the inflammatory response participates in all stages of prehepatic portal hypertension in the rat, not only during the initiation and first weeks of evolution, but also in the long-term stages. In this hypothetical situation, steatosis and dyslipidemia are thought to represent a common underlying factor of this syndrome, which features a chronic low-grade inflammatory state.

This chronic inflammatory state in the rat with portal hypertension could have splanchnic origin. In early evolutive stages, an increase in Fractalkine is produced in the mesenteric lymph nodes, associated with increased intestinal CX3CL1 [126]. Fractalkine (FKN/CX3CL1) is a chemokine that combines a dual function and acts as an adhesion and chemotactic molecule [154]. FKN is involved in the pathogenesis of numerous chronic inflammatory conditions including inflammatory bowel disease [155] and allergic asthma and rhinitis [156]. Considering that levels of pro-inflammatory cytokines are high in the mesenteric lymph nodes in portal hypertension, this could explain the increased production of FKN, with the recruitment of leukocytes and mast cells. Increased accumulation and activation of mast cells in the mesenteric lymph nodes could result in heightened and persistent chemokine production and mast cell recruitment, and therefore contribute to the chronicity of inflammation [85,86].

FKN could play a crucial role in the initiation and progression of inflammation in portal hypertensive rats. And so, the intestinal increase of CX3CL1, the unique receptor for FKN, is likely to be implicated in stimulating angiogenesis. FKN stimulates angiogenesis by activating the Raf-1/MEK/ERK and PI3K/Akt/eNOS/NO signal pathways via the G protein-coupled receptor CX3CR1 [157]. By this angiogenic activity, FKN could develop an important role in the pathogenesis of the angiogenesis-associated inflammatory process, which characterizes hypertensive enteropathy [81-83].

## Decompensation of the experimental portal hypertensive syndrome

Liver disease could be the most frequent factor for decompensating portal hypertension. Particularly, chronic liver disease and cirrhosis aggravate the portal hypertensive syndrome exceedingly.

The most studied models of cirrhosis in the rat are those achieved by extrahepatic cholestasis [44,158,159], by administration of carbon tetrachloride (CCl_4_) [44,160] or by administration of thioacetamide (TAA) [92,161]. Hepatic fibrogenesis is the common result of injury to the liver. Furthermore, fibrosis is believed to be a critical factor that leads to hepatic dysfunction [162].

Hepatic dysfunction related to fibrosis or cirrhosis in the rat would aggravate the grade of systemic inflammation characteristic of prehepatic portal hypertension and as a result would increase the incidence of complications. Consequently, the vascular dysfunction or hyperdynamic circulation with increased mesenteric blood flow would get worse [163,164] and intestinal lymph flow would be favored with and increased number of lymph vessels in the small bowel [163]. The incidence of ascites (44), renal failure [145], hepatopulmonary syndrome [142,165] and hepatic encephalopathy [166,167] would also increase.

The disturbance of splanchnic blood flow may contribute to an impairment of the intestinal barrier function and thus bacterial translocation is produced [168] with increased susceptibility to bacterial infections [158,159,168].

A decreased anti-oxidant capacity of the liver plays an important role in the pathogenesis of liver fibrosis or cirrhosis and portal hypertension [169-172]. That is why anti-oxidants have been proposed as an adjunctive therapy in the treatment of portal hypertension [170,172]. However, the deficient anti-oxidant capacity of the liver when suffering from fibrosis or cirrhosis could also induce the production of a systemic pathology. In this hypothetical situation, in prehepatic portal hypertensive rats with chronic oxidative stress and a low-grade inflammatory state, the reduction of the hepatic anti-oxidant capacity would increase the intensity of the inflammatory systemic response and add severity to this syndrome. Therefore, the relationship between the liver anti-oxidative capacity and the severity of the systemic complications could be more important than the grade of splanchnic and systemic oxidative stress. Aside from the degree of oxidative stress, the reduction of the hepatic anti-oxidant capacity would aggravate the intensity of the inflammatory response [18].

It is possible that another organ, like the endothelium, associated with the progressive reduction of the anti-oxidant capacity of the liver in the evolution of cirrhosis, tries to make up for the deficit. In this case, the objective of angiogenic systemic hyperactivity could be to reduce oxidative and enzymatic stress associated with inflammation [18].

## Anti-inflammatory angiogenesis and chronic liver disease

Mammals, along with other aerobic organisms, have evolved an array of mechanisms to protect themselves from the potential harmful effects of reactive oxygen species [173]. Oxidants are products of a normal aerobic metabolism and the inflammatory response [173], so their formation can't be avoided. The formation of reactive oxygen species is, therefore, prevented by an efficient anti-oxidant system made up of a group of compounds with different properties and mechanisms [173,174]. These include enzymes, such as catalases, peroxidase and superoxide dismutase, and repair enzymes, such as DNA glycosylases, as well as water and lipid-soluble anti-oxidants such as ascorbic acid (vitamin C), α-tocopherol (vitamin E) and β-carotene [173,174]. Other molecules that also have anti-oxidant properties are glutathione [174] and albumin [175,176].

Multiple enzymes expressed in vascular cells are involved, not only in the production but also in the elimination or scavenge of reactive oxygen species, including superoxide dismutases, catalase, thioredoxin reductase, glutathion, peroxidase, NAD(P)H oxidase, xanthine oxidase, myeloperoxidase and endothelial oxide synthase [177]. Anti-oxidants can modulate endothelium-dependent vasodilation responses, the balance between pro- and anti-thrombotic properties, the homeostatic endothelium leukocyte interactions and the vascular apoptotic responses [178]. All of these functions are altered in the cirrhotic stage [62,75]. That is why it can be considered that chronic liver disease has a type of "endothelial dysfunction." This term has been used to refer to a number of pathological conditions involving the vascular endothelium, for example, impairment of endothelium-dependent vasorelaxation, altered anticoagulant-antithrombotic functions, anti-inflammatory properties of endothelium and impaired modulation of vascular growth with deregulation of vascular remodeling [75,179]. This group of alterations have been described in clinical and experimental cirrhosis [41,75]. They are associated with portal hypertension and [5,48,59,62], in essence, make up the pathophysiological mechanisms that play the leading role in the evolutive phases of the inflammatory response [12-15].

In the cirrhotic stage, the impaired modulation of vascular growth with deregulation of vascular remodeling is a pathophysiological mechanism that not only participates in the production of splanchnic alterations (cirrhotic liver, splenomegaly, enteropathy, portosystemic collateral circulation) but also in different systemic alterations (hepatic encephalopathy, hepatopulmonary syndrome, portopulmonary hypertension, vascular spiders, digital clubbing) [125,140,180,181]. The angiogenic response in chronic liver disease contributes significantly to structural splanchnic and systemic remodeling. Under physiological conditions, endothelial cells are normally quiescent. They replicate at a very slow rate. However, in pathological situations, endothelial cells can proliferate rapidly with a turnover time of less than 5 days [179].

Angiogenesis associated with inflammation when the anti-oxidant capacity of the cirrhotic liver fails could also reflect the establishment of a substitute anti-oxidant mechanism, which would explain its excessive response and the extensive diffusion. The anti-oxidant, anti-enzymatic and anti-inflammatory properties of endothelium [178] allow for suggesting that angiogenesis is a defensive mechanism when the liver fails to produce anti-oxidant molecules due to cirrhosis. In this sense, perhaps it may be interesting to remember that the origin of vasculogenesis relies on the yolk sac during embryonic development [182]. In the embryo, the blood islands consist of hematopoietic cells surrounded by endothelial cells and form the distal part of the yolk sac. These endothelial cells of the blood islands expand to cover the entire yolk sac forming a vascular network, known as the capillary plexus [182]. Interestingly enough, the yolk sac membrane is a highly vascularized structure that transfers lipids from the yolk sac to the embryo [183].

## Inflammatory phenotypes in chronic hepatic disease in the cirrhotic patient

The study of experimental prehepatic portal hypertension and its decompensation when associated with "hepatic failure" offers results that could be extrapolated with caution to the evolution of patients with chronic liver disease related to cirrhosis [38,39]. At the same time, the evolution and complications that these patients suffer suggest the participation of the mechanisms characteristic of the inflammatory response in their pathogeny. That is why three pathological phenotypes could be distinguished during the evolution of chronic hepatic failure in human clinical trials.

• Ischemia-revascularization phenotype, which has hemodynamic alterations and oxidative and nitrosative stress

• Leukocytic phenotype with predominant enzymatic stress and acute phase inflammatory response.

• Angiogenic phenotype, that evolves early, and whose objective is tissue remodeling

## Ischemia-Revascularization phenotype

Splanchnic venous stasis related to increased intrahepatic resistance could be the initiating factor of this phenotype. This would be the origin of reflex responses within the brain-splanchnic axis, mediated by the autonomic nervous system, the renin-angiotensin-aldosterone system and the hypothalamic-pituitary-adrenal axis [34,41]. The activation of these systems would explain most of the hyperdynamic alterations related to splanchnic venous stasis and therefore, also related to hypoxia, which imposes blood stasis on the organs and tissues that drain the splanchnic venous system [18,62].

The hyperdynamic circulatory syndrome that is produced in chronic liver diseases has recently been called "Progressive Vasodilatory Syndrome" because vasodilation is the factor that brings about all the vascular changes and finally leads to the multi-organ involvement observed as a consequence of this hemodynamic change [56,184].

The mechanisms promoting vasodilation in the Progressive Vasodilatory Syndrome are complex [56,184]. However, most of the mediators involved in their production are shared by other systemic vasodilatory conditions as for example, congestive heart failure and vasodilatory shock [185-187]. This vasomotor systemic response is common to several pathological conditions, and it has been proposed that it could represent the first phase of the systemic inflammatory response, since the establishment of an ischemia-reperfusion phenomenon with blood flow redistribution would be reflected [6,12-14].

In polytraumatized patients, prolonged and severe hypotension are also the cause of vasodilatory shock [186] with related or late multiple organ dysfunction or failure [22,188]. Interestingly, it has also been suggested that the gastrointestinal tract often represents the source for the development of related multiple organ failure [189].

**Figure 9:**
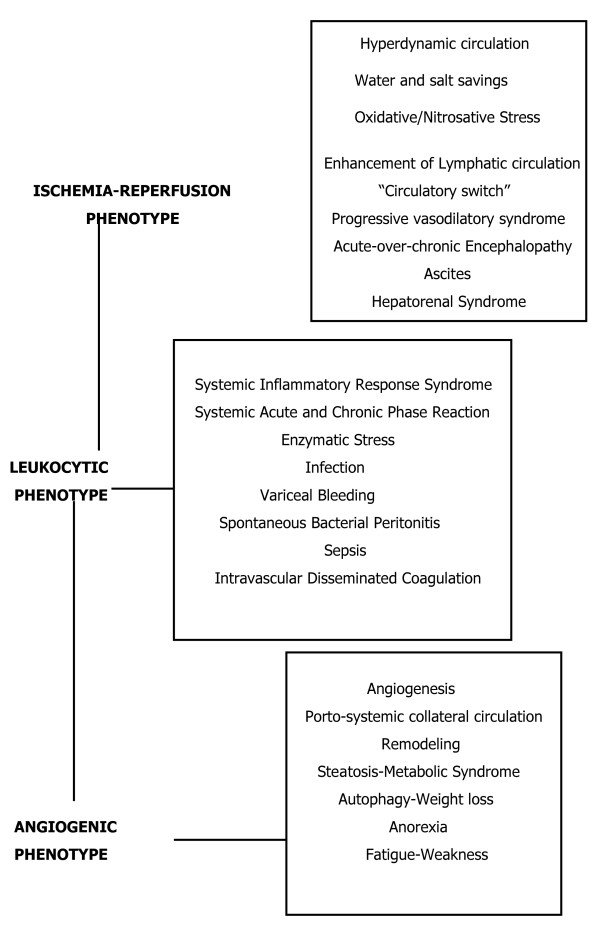
Inflammatory phenotypes in the evolution of chronic liver disease.

During the evolution of chronic hepatic disease, the factors that produce its decompensation and aggravate hypoxia [1,190,191] (acute-over-chronic hepatic failure) are also inducers of the hyperexpression of the ischemia-revascularization phenotype. Thus, the hepatorenal syndrome is produced, which is characterized by sodium and water retention with renal vasconstriction, resulting in decreased renal blood flow, glomerular filtration rate, and urinary output, which contribute to azotemia [39]. Another major complication includes ascites [192]. The ascitic fluid total protein level typically has been used in defining ascitic fluid as transudative (protein content less than 2.5 g per dL) or exudative (protein content of 2.5 g per dL or greater) [39](Figure 9).

## Leukocytic phenotype

The alterations associated with this phenotype have driven experts in chronic hepatic disease to support the inflammatory nature of this disease [193-195].

The immune response underlying the expression of the leukocytic phenotype could also have a gastrointestinal origin. The gastrointestinal tract mucosa contains the largest reservoir of macrophages in the body. As effector cells, intestinal macrophages, together with mast cells [86,87] are part of the first-line defense mechanisms [196]. These first-line mechanisms represent an ancient defense system that arose perhaps a billion years ago in early multi-cellular organisms and are still used today in protozoa, insects, plants and mammals [197]. Resident intestinal macrophages do not express innate response receptors but in the inflamed mucosa, display a different phenotype and functional pro-inflammatory profile [196]. Also epithelial cells could be involved in the initiation and propagation of intestinal inflammation in response to pathophysiological stimuli in the cirrhotic patient since they alter the permeability of the mucosa barrier [198]. The activation of the splanchnic endothelium system by hypoperfusion/hypoxia [190] would aggravate intestinal epithelium injury and may favor the release of pro-inflammatory mediators that can amplify the Systemic Inflammatory Response Syndrome.

At the same time, a compensating response is produced through the induction of a systemic acute and chronic phase reaction, where the liver and intestine mainly participate [199-202]. In this response, positive acute phase proteins are produced which have the following properties: anti-oxidant (scavenging free radicals); anti-enzymatic (α_1_-anti-trypsin and α_1_-anti-chymotrypsin) and anti-bacterian (opsonization and trapping of micro-organisms and their products) properties [203].

If this defense capacity of the Systemic Acute and Chronic Phase Reaction is overtaken, the intestine, as in the critically ill surgical patient, becomes an "undrained abscess" [204,205] and the pathological gastrointestinal colonization is associated with the development of infection [39,193], sepsis and disseminated vascular coagulation [206-208]. Also during the hyperexpression of this immune response the lymphatic circulation would acquire increasing importance and in the mesenteric lymph nodes, cells able to present antigens (dendritic cells, macrophages and mast cells), would broaden or modulate the systemic splanchnic inflammatory response [18] (Figure 7).

## Angiogenic phenotype

Angiogenesis is defined as the growth of new vessels from preexisting ones [209]. Although the final objective of endothelial growth is to form new vessels for oxygen, substrates and blood cells (vascular phase) other functions could also be carried out before the new vessels are formed (prevascular phase).

In the initial phases of the inflammatory response, the new endothelial cells formed could have a function associated with anti-inflammatory effects. That is, with anti-oxidative and anti-enzymatic stress properties, favoring the resolution as well as the progression of the inflammation [18].

Angiogenesis is essential for embryogenesis, tissue growth and tumorigenesis. Also, it is been found to be central to the progression of various chronic inflammatory conditions including chronic hepatic disease [62,182,209]. In particular, when inflammation is produced, endothelial proliferation begins early and is controlled by a wide variety of positive and negative regulators, which are composed of neurotransmitters, cytokines, chemokines, adhesion molecules and growth factors [210]. Therefore, all the mediators that characterize the three proposed phases of the inflammatory response are regulators of the endothelial growth. The tight overlapping between the inflammation mediators and the newly formed endothelial cells could reflect the functional importance of these last phases in the progression of the inflammation. There is considerable evidence to suggest that angiogenesis and chronic inflammation are codependent [211].

In chronic hepatic disease, endothelial proliferation could be associated with anti-inflammatory effects. In this hypothetical situation, endothelial growth would represent an ancient mechanism that the body uses to protect cellular structures against oxidative and enzymatic stress [212]. This could mean the relation between angiogenesis, non alcoholic fatty liver disease and metabolic syndrome in portal hypertension.

Angiogenesis is critically dependent on the VEGF action, but VEGF also plays a critical role in macrophage recruitment and infiltration. Also, in concert with angiopoietin 1, VEGF may act to help maintain vascular integrity in adipose tissue in a paracrine manner [213]. Therefore, in lipid accumulation (metabolic switch), considered pathological, a defense mechanism could arise that reduces the harmful effects of oxidative stress in the body [214]. If endothelial growth and intracellular lipid accumulation are considered effective anti-oxidant mechanisms, their inhibition in different pathological processes, including portal hypertension, could have detrimental results if they are not associated with an efficient anti-oxidant therapy substitute. So, lipid replacement therapy administered as a nutritional supplement with anti-oxidants can prevent excess oxidative membrane damage, restore mitochondrial and other cellular membrane functions and reduce fatigue [215].

This precarious balance between oxidative/enzymatic stress and anti-oxidant/anti-enzymatic abilities that could characterize chronic liver disease, is difficulty decompensated. Mainly the liver, due to its important anti-oxidant/anti-enzymatic capacity when suffering functional damage from fibrosis or cirrhosis, would aggravate the complications characteristic of portal hypertension and consequently, would increase morbidity and mortality [18].

Factors that are secreted mainly from the liver counteract obesity and related insulin resistance, acting as endocrine signals in the peripheral tissues to regulate metabolic homeostasis [216]. On the contrary, its deregulation as well as the increased levels of angiopoyetina 13, might be involved in inducing hypertriglyceridemia and insulin-resistance [215]. Therefore, hepatocyte-derived circulating factors that regulate lipid metabolism might be involved in the pathogenesis of the metabolic syndrome in portal hypertension. It is important that angiopoietins play roles not only in lipid metabolism, but also in hematopoiesis and in angiogenesis [212,213,215], three functions that are successively expressed by the liver during its embryonic development.

Angiogenesis participates actively in the remodeling process that cirrhotic patients suffer, in which macrocirculatory (portosystemic collateral circulation) and microcirculatory changes are produced in all tissues and organs of the body. Increasing the catabolism of glycogen, adipose fat and muscle proteins, the redistribution of materials for remodeling is achieved. In this organic restructuring, there could be more autophagic activity [217]. Autophagic lipolisis and proteolisis would allow for getting materials for the disproportionate systemic angiogenesis, although at a high cost for the normal functioning of the body. Thus, the patients, even in an early and well-compensated stage of cirrhosis, can manifest anorexia and weight loss, weakness and fatigue [38] (Figure 7).

These three phenotypes, ischemia-revascularization, leukocytic and angiogenic, could represent the pathological functions that are predominantly expressed during the evolution of chronic liver disease. If the three phenotypes are compared to the three pathological systemic functions suggested to make up the systemic inflammatory response [12-15], it could also be considered that they constitute increasingly complex trophic functional phenotypes. Thus, during the expression of the ischemia-revascularization phenotype, a savings in energy and sodium through hydration would be produced, which enhances nutrition by diffusion (nervous functional system). The leukocytic phenotype would favor tissue nutrition mediated by leukocytes through symbiosis with bacteria (immune functional system) and, finally the objective of the angiogenic phenotype would be to reestablish nutrition mediated by blood capillaries (endocrine functional system). Hence, the successive expression of these phenotypes of increasingly trophic functions during the evolution of chronic liver disease would constitute the phenotypes characteristic of a chronic systemic inflammatory response. In this hypothetical situation, the incidence of harmful influences during their evolution could involve regression to the most primitive trophic stages, where nutrition by diffusion (ischemia-revascularization phenotype or functional nervous system), which is simpler but also less costly, facilitates temporary survival until a more favorable environment makes it possible to initiate more complex nutritional methods (leukocytic phenotype or functional immune system and angiogenic phenotype (functional endocrine system) [13,15]. Perhaps this is the reason why the decompensation of cirrhotic patients results in complications linked to the ischemia-revascularization phenomenon with oxidative stress and edema, for example acute chronic hepatic encephalopathy, ascites and hepatorenal syndrome [39].

Hepatic fibrogenesis is the common result of injury to the liver. This process is progressive and leads to hepatic dysfunction. In particular, the incapacity of the liver to provide the body anti-oxidant factors when the organism returns to the metabolic stages characterized by a deficient use of oxygen (ischemia-revascularization phenotype) would prevent the progression of the inflammatory response and therefore would favor the persistence of the metabolic regression with progressive worsening of the mentioned complications [18].

Since the phenotypes of chronic liver disease, like the phases described for post-traumatic inflammation [13-15] go from ischemia to a progressive oxygenation, it is also tempting to speculate on whether the body reproduces some of the successive stages by which life passes from its origin without oxygen until it develops an effective, although costly, system for the use of oxygen [218]. If so, the successive metabolic switches that the body suffering chronic liver disease undergoes, allows it to survive until a more favorable environment makes it possible to initiate a more complex oxidative metabolism. The hypothesized capacity of the body to involute, dedifferentiate or return to early stages of development could constitute an effective defense mechanism against injury since it would make it possible to retrace a well known route, which is, the prenatal specialization phase. However, it has the disadvantage that it tries to develop its morphofunctional specialization although the aggression from harmful factors is not interrupted. Meanwhile, efficient anti-oxidant mechanisms are established (portal hypertension, cirrhotic liver) without the functional support of the placenta [219].

The persistence in the expression of old metabolic states, linked to the deficient use of oxygen, could be associated with the accumulation of metabolites that in ancient evolutive states favored life. Today, some of these metabolic mechanisms are still used by fishes, amphibians and reptiles to survive the extremes of oxygen availability [220]. And so, it has been proposed that our species evolved under "colder, drier and higher" conditions and that is why these adaptations may represent the "ancestral" physiological condition for humans [221,222]. Therefore, in portal hypertension and chronic liver disease, the metabolic alterations that are produced could have been beneficial in the past. For example, the predominance of the lipid metabolism with the accumulation of cholesterol, a precursor molecule of many hormones like progesterone, corticoids, aldosterone, androgens and estrogen; the establishment of ancient anti-oxidant mechanisms, like sulphydryl compounds, hydrogen sulfide (H_2_S) and glutathione [223] and the heme-oxigenase 1 system [224]; the hyperproduction of NH_4, _a prebiotic metabolite, and its relation to the ancient use of the electron acceptor N to reduce this gas to NH_3 _[225]; or the hyperactivity of the fermentation pathways associated with insulin resistance [106].

The progressive specialization in the use of oxygen can be considered one of the pathways for understanding the successive metabolic stages that play leading roles in life on earth from its anaerobic origin through today. The hypothesis that atmospheric oxygen concentrations affected the timing of the evolution of cellular compartmentalization by constraining the size of domains necessary for communications across membranes has been suggested [226]. This points towards a key role for oxygen in the increased abundance and size of receptors over time [226]. It also adds to a growing body of literature connecting atmospheric oxygen levels with macroevolutionary changes, most recently with complexity in metabolic networks and cell types [226,227].

In summary, the pathology considered to be the expression of ancestral biochemical functional systems could support the information needed for better understanding how life evolved on earth, mainly involving five elements: hydrogen, carbon, nitrogen, sulfur and oxygen [225,228].

## Authors' contributions

All the authors conceived, discussed, wrote and approved the manuscript.
